# The Book World of Medicine and Science

**Published:** 1894-01-13

**Authors:** 


### The Book World of Medicine and Science.

### THE ENDOWMENT OF PUBLIC PRACTITIONERS.—I.*

Candidates for a commission in the Army Medical Staff must be young men of unmixed European blood, between the ages of 21 and 28 years, and possessed of a double qualification. A recent ordinance (March 31st, 1893) insists that they shall also have acted for six months as clinical clerk and six months as surgical dresser, not less than three months of each term having been spent in the wards of a hospital. They must also have attended a three months' course of ophthalmic instruction. Although possessed of these certificates and diplomas, a candidate must undergo another examination in professional subjects, while French German, and natural sciences are recommended as voluntary subjects, and the two languages are specially desired. After this examination is passed, the candidate is regarded as a surgeon on probation, and as such attends a course of study at the Army Medical School at Netley bearing more directly on military work. During this time he receives pay at the rate of 8s. per day.

When his studies are completed he ranks as surgeon-lieutenant, and receives a salary of £200 a year. He may be promoted within three years to be surgeon-captain, but this implies no increase of pay until he has completed five years' service, when he receives £250 a year. A surgeon-major, promoted from among the surgeon-captains on the recommendation of the director-general of the Army Medical Department, after twelve years' service, of which three have been spent abroad, receives £650 per annum for pay and allowances, or for pay exclusive of allowances £1 a day, increased to £1 2s. 6d. after fifteen years' service. A surgeon-lieutenant-colonel receives the same yearly inclusive salary; but if he takes the daily pay without allowances he receives £1 5s. after twenty years' service, and £1 7s. 6d. after twenty-five. The next grade is that of brigade-surgeon-lieutenant-colonel whose yearly salary is £750, and daily pay without allowance £1 10s., rising to £1 13s. after he has held the rank for five years. The brigade-surgeon-lieutenant-colonel is selected on the recommendation of the commander-in-chief, and must have served abroad for at least eight years. For the next rank, surgeon-colonel, no one is eligible who has not served abroad for at least ten years, of which three must have been spent in India. A surgeon-colonel's inclusive pay is £900 per annum, or without allowances £2 a day. He, too, must be recommended by the Commander-in-Chief, the grounds of the recommendation to be expressly stated in writing. The highest position in the service is that of Director-General of the Army Medical Department, the salary attached to which is £1,500 a year.

The retired pay varies according to length of service as well as to rank. A director-general, who has filled that post for seven years and has been over thirty years in the Department, is entitled to retired pay of £1,125 a year. If he has been for five years director-general he receives £1,000, and if less than five £875. If his retirement takes place after only three years in the post, and less than thirty years in the service, he receives the retired pay of a surgeon-major-general, plus £100 a year. The appointment of director-general is made for seven years; then the occupant is compelled to retire. For other ranks the age of retirement is, for surgeon-major-general and surgeon-colonel, sixty; for all below, fifty-five.

A surgeon-major-general on retiring receives £2 a day, and a surgeon-colonel £1 15s. A brigade-surgeon-lieutenant-colonel who retires after thirty years' service has £1 10s. a day; if he has served a shorter period his pay is only £1 7s. 6d. Similarly a surgeon-lieutenant colonel who has not served more than twenty years gets £1 a day retired pay, if he has twenty-five years' service £1 2s. 6d., and if thirty years an additional half-crown a day is put on. Medical officers with less than twenty years' service do not receive a pension, but only a gratuity on retiring. This amounts, in the case of those officers from surgeon-lieutenant-colonel downwards, who have served only ten years, to £1,250; fifteen years' service entitles them to £1,800, and eighteen years to £2,500. Six of the most distinguished officers of the Army Medical Staff are appointed honorary physicians to the Queen and six honorary surgeons.

The Indian Medical Service resembles the Home Army Medical Staff in almost all points. It, however, accepts any natural born subject of the Queen, even though not of pure European extraction. After passing the preliminary examination, the surgeon on probation receives 10s. a day while he is at Netley, and either goes to India in a troopship, or has his passage paid in an ordinary vessel. On first appointment he receives 317 rupees 8 annas a month, or, approximately £255 a year. When appointed to the charge of a native regiment, he usually receives a consolidated salary (in lieu of pay and staff allowance) of 450 rupees a month, with 60 rupees for the keep of a horse if the regiment is a cavalry one, in all about £420 a year. A surgeon-captain with more than five years' service, is paid 600 rupees a month with the same horse allowance (£552 per annum), and a surgeon-major, if he has less than twenty years' service, 800 rupees a month and 90 rupees horse allowance (£780 per annum). A brigade-surgeon-lieutenant-colonel of twenty years' service or more, has a salary of 1,000 rupees a month, with the same horse allowance as the surgeon-major. Of the higher appointments, a surgeon-colonel receives from 1,800 to 2,250 rupees a month (£1,440 to £1,800 per annum); a surgeon-major-general in Madras or Bombay, about £2,000 a year, and in Bengal 2,700 rupees a month, equal to £2,160 yearly. These emoluments may, however, be increased in various ways. Thus, if an officer is acting in another appointment than that which he holds permanently, he receives (1st) the pay, but without allowances of his own rank; (2nd) half the staff allowance of his own rank; (3rd) half the staff allowance of the higher rank in which he is officiating. To obtain this, however, it is necessary to pass an examination in Hindustani. Medical officers are eligible for appointments in the Assay Department at salaries varying from 690 rupees to 2,250 rupees a month (£480 to £2,019 per annum). Pay while on leave out of India is granted at the rate of £200 a year on first appointing, increasing £50 with every five years' service, till after the commencement of the first year's service it reaches £500 a year. With regard to pensions, seventeen years' service is required to qualify for one, which then amounts to £292 per annum. After twenty years' service the pension is £365 a year, after twenty-five, £500; and after thirty years, £700. Officers not entitled to pensions receive the British rate of half-pay of their rank. The widows and families of officers receive pensions at the same rate as those of British officers, and are also entitled to pensions from the Indian Service Family Pension Fund, to which all officers are compelled to contribute.

* See Faulkner's "Guide to the Public Medical Services." (London: H. K. Lewis, 136, Gower Street. Demy 8vo. Two Shillings.)

